# Molecular analysis of knockdown resistance (*kdr*) mutations in the voltage-gated sodium channel gene of *Aedes aegypti* populations from Saudi Arabia

**DOI:** 10.1186/s13071-022-05525-y

**Published:** 2022-10-19

**Authors:** Abadi M. Mashlawi, Ashwaq M. Al-Nazawi, Elsiddig M. Noureldin, Hussain Alqahtani, Jazem A. Mahyoub, Jassada Saingamsook, Mustapha Debboun, Martha Kaddumukasa, Hesham M. Al-Mekhlafi, Catherine Walton

**Affiliations:** 1 Department of Earth and Environmental Sciences, Faculty of Science and Engineering, University of Manchester, Manchester, M13 9PL UK; 2 Department of Biology, Faculty of Science, Jazan University, Jazan, 45142 Saudi Arabia; 3 Preventive Medicine Department, Public Health Directorate, Ministry of Health, Jeddah, Saudi Arabia; 4 Saudi Center for Disease Control & Prevention, Ministry of Health, Jazan, Saudi Arabia; 5 Department of Biology, Faculty of Science, University of Tabuk, Tabuk, Saudi Arabia; 6 Department of Biology Sciences, Faculty of Science, King Abdulaziz University, Jeddah, Saudi Arabia; 7 Center of Insect Vector Study, Department of Parasitology, Faculty of Medicine, Chiang Mai University, Chiang Mai, 50200 Thailand; 8 Delta Mosquito and Vector Control District, Visalia, CA USA; 9 Department of Biological Sciences, Faculty of Science, Kyambogo University, P.O. Box 1, Kampala, Uganda; 10 Department of Epidemiology, Faculty of Public Health and Tropical Medicine, Jazan University, Jazan, 45142 Saudi Arabia; 11 Department of Parasitology, Faculty of Medicine and Health Sciences, Sana’a University, P.O. Box 1247, Sana’a, Yemen

**Keywords:** *Aedes aegypti*, Insecticide resistance, Knockdown resistance, *kdr*, Mutations, Median-joining haplotype network, Haplotype, Saudi Arabia

## Abstract

**Background:**

The *Aedes aegypti* mosquito is the primary vector for dengue, chikungunya, yellow fever and Zika viruses worldwide. The first record of *Ae. aegypti* in southwestern Saudi Arabia was in 1956. However, the first outbreak and cases of dengue fever were reported in 1994, and cases have increased in recent years. Vector control for *Ae. aegypti* mainly uses pyrethroid insecticides in outdoor and indoor space spraying. The constant use of pyrethroids has exerted intense selection pressure for developing target-site mutations in the voltage-gated sodium channel (*vgsc*) gene in *Ae. Aegypti* against pyrethroids—mutations that have led to knockdown resistance (*kdr*).

**Methods:**

*Aedes aegypti* field populations from five regions (Jazan, Sahil, Makkah, Jeddah and Madinah) of southwestern Saudi Arabia were genotyped for known *kdr* mutations in domains IIS6 and IIIS6 of the *vgsc* gene using polymerase chain reaction (PCR) amplification and sequencing. We estimated the frequency of *kdr* mutations and genotypes from Saudi Arabia as well as from other countries, Thailand, Myanmar (Southeast Asia) and Uganda (East Africa). We constructed haplotype networks to infer the evolutionary relationships of these gene regions.

**Results:**

The three known *kdr* mutations, S989P, V1016G (IIS6) and F1534C (IIIS6), were detected in all five regions of Saudi Arabia. Interestingly, the triple homozygous wild genotype was reported for the first time in two individuals from the highlands of the Jazan region and one from the Al-Quoz, Sahil region. Overall, nine genotypes comprising four haplotypes were observed in southwestern Saudi Arabia. The median-joining haplotype networks of eight populations from Saudi Arabia, Southeast Asia and East Africa for both the IIS6 and IIIS6 domains revealed that haplotype diversity was highest in Uganda and in the Jazan and Sahil regions of Saudi Arabia, whereas haplotype diversity was low in the Jeddah, Makkah and Madinah regions. Median-joining haplotype networks of both domains indicated selection acting on the *kdr*-mutation containing haplotypes in Saudi Arabia.

**Conclusions:**

The presence of wild type haplotypes without any of the three *kdr* mutations, i.e. that are fully susceptible, in Saudi Arabia indicates that further consideration should be given to insecticide resistance management strategies that could restore pyrethroid sensitivity to the populations of *Ae. aegypti* in Saudi Arabia as part of an integrative vector control strategy.

**Graphical Abstract:**

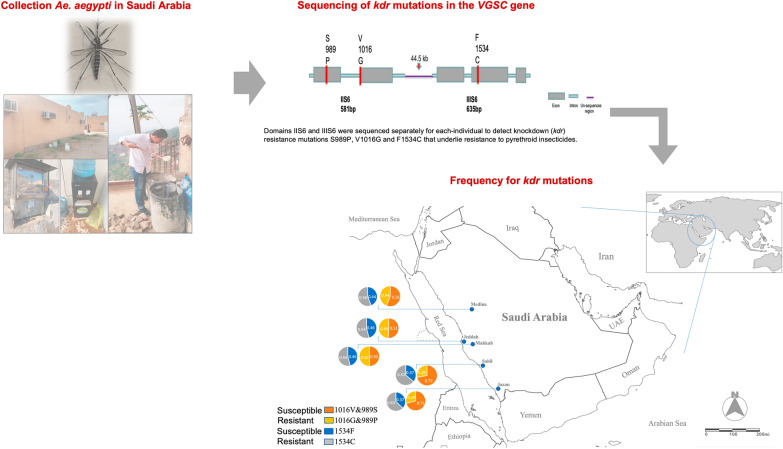

**Supplementary Information:**

The online version contains supplementary material available at 10.1186/s13071-022-05525-y.

## Background

The mosquito *Aedes aegypti* is responsible for transmitting life-threatening infectious arboviruses, including dengue, chikungunya, yellow fever and Zika [1]. This mosquito has spread from Africa to other tropical and subtropical regions of the world, resulting in a significant increase in their burden of vector-borne diseases [2, 3]. *Aedes aegypti* was reported in the Arabian Peninsula, including Saudi Arabia, in 1956 [4]. The form of *Ae. aegypti* in Saudi Arabia has been genetically characterised as the domestic form that spread out of Africa [5, 6]. However, the first dengue fever outbreak was reported in the 1990s [7], and in more recent years, the dengue incidence rate increased to 21.71 per 100,000 persons/year in 2013, and then dropped to 11.59 in 2019 [8, 9]. Dengue fever cases are concentrated in the country’s southwest regions, i.e. Jazan, Sahil, Jeddah, Makkah and Madinah [9–11]. There was one confirmed case of chikungunya in 2011 [12] and another in 2021 [13], but no reported cases to date of Zika virus.

Although new vector control strategies are in development (i.e. genetic manipulation and *Wolbachia*-based population control), controlling *Ae. aegypti* using insecticides remains the primary method applied, especially during an outbreak [14]. In Saudi Arabia, insecticide usage for vector control has been documented since 1948 due to endemic malaria [15, 16]. Various insecticides have been used in the country: dichlorodiphenyltrichloroethane (DDT) was used from 1948 to 1954 and from then, other insecticides were used instead [16]. The first report of pyrethroid resistance was in 2011 in Makkah [17], followed by Jeddah [18] and Jazan [19]. Pyrethroids have been used mainly against *Ae. aegypti* both publicly by the government [18, 19] and privately by house owners [20]. The widespread use of pyrethroids worldwide has been due to their lower environmental effects, low mammalian toxicity and, most importantly, their fast action on the target pest [21]. To date, pyrethroids are the only recommended insecticides for indoor application [22]. With the overuse of insecticides, mosquitoes, particularly *Ae. aegypti*, have developed resistance worldwide to multiple insecticides, making them extremely difficult to control [14, 23].

DDT and pyrethroid insecticides are neurotoxins that deliver toxic effects by binding to the voltage-gated sodium channel protein (VGSC). The binding of insecticides to the sodium channel causes a change in its gating properties and keeps it open for a long time, which eventually causes death [24]. The VGSC-encoding gene (*vgsc*) is large in insects, with a single copy encoding for a protein (about 2050 amino acids) [24]. In *Ae. aegypti*, the *vgsc* comprises 37 exons with a length of approximately 480 kb (VectorBase). Several non-synonymous mutations in the *vgsc* that cause knockdown resistance (*kdr*) are associated with pyrethroid and DDT resistance [23–25]. These mutations (S989P, V1016G and F1534C) were reported in the Middle East and Southeast Asia [25]. *Kdr* mutations at amino acid 1016 have been reported from different geographical locations; for example, V1016G in Southeast Asia since 2009 [26–33], Ghana [34] and Saudi Arabia [17, 35]. The mutation S989P is thought not to cause any resistance alone but when co-occurring with V1016G confers resistance against permethrin and deltamethrin [17]. The F1534C mutation is found in Asian, American and African populations of *Ae. aegypti* [36, 37].

Frequencies of *vgsc* genotypes involving the three *kdr* mutations (S989P, V1016G and F1534C) vary among locations. To date, eight and 12 genotypes have been reported from Saudi Arabia and Myanmar, respectively, while Thailand has reported only three [30, 35, 38]. The genotype that is homozygous for mutations S989P and V1016G but is wild type at F1534C has a high resistance level to both type I (i.e. permethrin) and type II (i.e. deltamethrin) pyrethroids [35]. In contrast, the genotype (S989P, V1016G wild type with F1534C homozygous mutant, i.e. SS + VV + CC) confers low resistance to type I pyrethroids [17, 39, 40]. Additionally, intermediate (in type I and II pyrethroids) and nearly susceptible levels of resistance (in type II pyrethroids) were observed in the fully heterozygous genotype SP + VG + FC and the partially heterozygous genotype SP + VG + CC [39]. The genotype that is only heterozygous for a *kdr* mutation at position 1534 (SS + VV + FC) had a slightly elevated resistance level compared with the wild type (in type I pyrethroids) [39]. In a previous study in Saudi Arabia (2017), the resistance status of genotypes PP + GG + FC and SP + VG + CC was not completely ascertained, but they were perhaps susceptible (in type II pyrethroids) [17]. Finally, the triple homozygous mutant genotype PP + GG + CC confers a much higher resistance level when expressed in *Xenopus* oocytes [40]. However, this genotype is rarely observed in natural populations of *Ae. aegypti* [30, 35]*.*

For effective vector control campaigns, it is essential to detect *kdr* mutations and characterise their frequencies and distributions. Further, understanding the evolution of *kdr* mutations may help us in managing insecticide resistance. Within this context, this study aimed to (i) detect and investigate the frequency of *kdr* mutations in five dengue fever-endemic regions of Saudi Arabia, (ii) characterise the genotypes and haplotypes within Saudi Arabia and assess whether they are shared with other populations worldwide, and (iii) construct putative evolutionary relationships amongst sequences to infer the dispersal and origin of *kdr* haplotypes in Saudi Arabia.

## Methods

### Study area

**Fig. 1:**
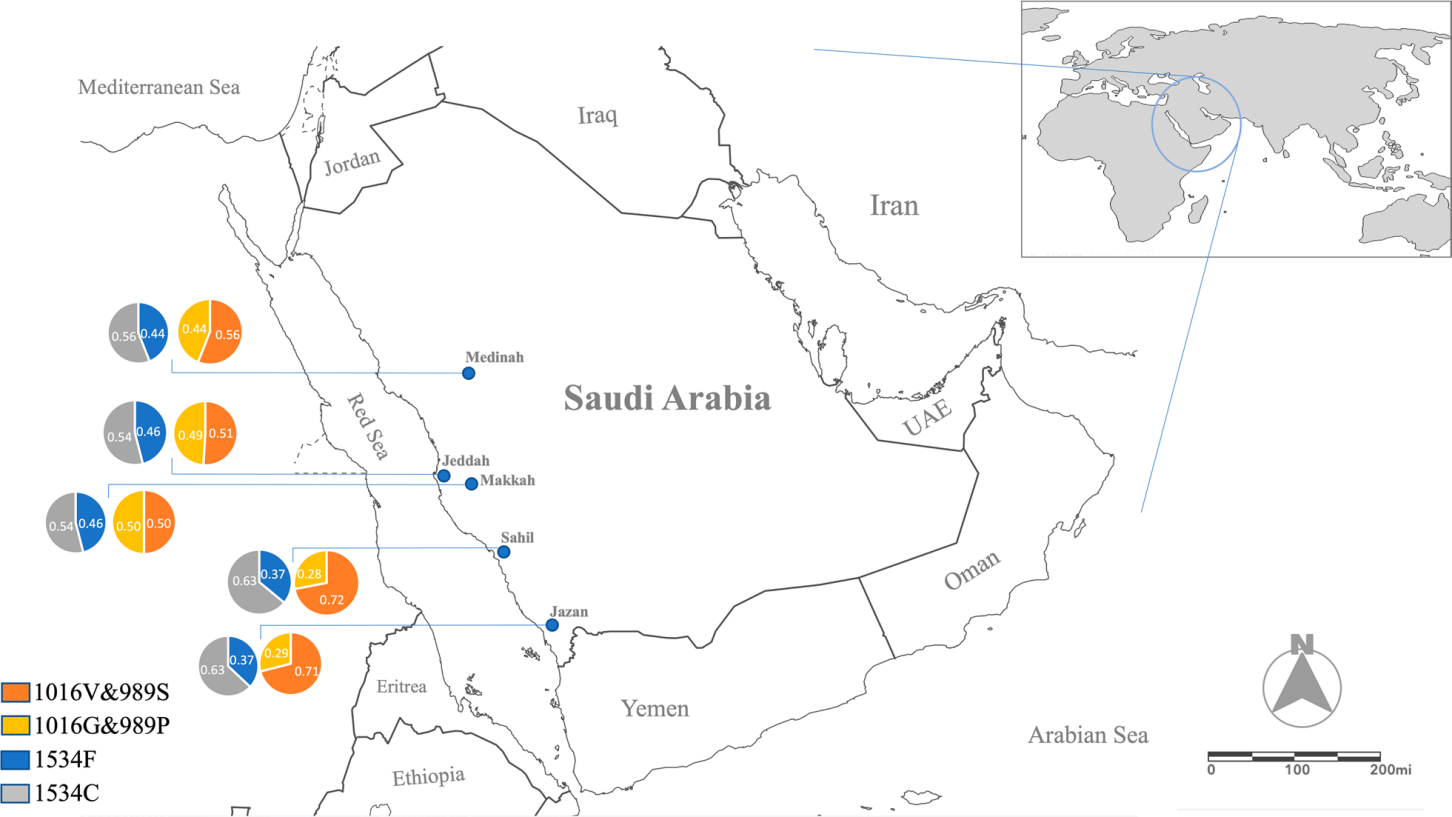
Map of Saudi Arabia showing the five sampling regions of *Ae. aegypti*, i.e. Jazan, Sahil, Makkah, Jeddah and Madinah with frequency for *kdr* mutations S989P + V1016G and F1534C observed in this study. ArcGIS website was used to generate the map

Saudi Arabia, which lies in Western-South Asia, is the largest country in the Arabian Peninsula, occupying a total land area of about 2,000,000 km^2^ and a total population of about 35 million [41]. Geographically, Saudi Arabia is divided into three distinct zones: the rain-fed highlands of the western and southwestern regions (Sarawat Mountains), the arid and extra-arid lands of the interior (Najd) and the coastal plain along the Red Sea in western Saudi Arabia (known as the Tihamah) that includes the east of the plain represented by the Hejaz and the Asir mountain range. Five different regions of Saudi Arabia, namely, Jazan, Sahil, Jeddah, Makkah and Madinah were selected for this study (Fig. 1 and Additional file 1: Table S1).

The Jazan region is located in the southwestern part of the country (16°53’N, 42°34’E) alongside the Red Sea and shares a border with Yemen to the south (120 km), where it is known for endemic vector-borne diseases such as dengue fever and malaria [42]. Jeddah and Makkah (21°32′N, 39°10′E and 21°25′N 39°49′E) are about 700 km north of Jazan. The Jeddah region (with a population of 3.4 million) is considered to be the area with the second-largest economy in Saudi Arabia, after the capital city (Riyadh), which makes it attractive for migrant workers from all over the world [43]. Furthermore, Jeddah has the largest and busiest port (Jeddah Islamic Port) in the Middle East and North Africa [44]. Every year, over two million people gather from across the world, including South Americans, Africans, Europeans and Asians, in Makkah (arriving through the international airport in Jeddah) to practise Islamic pilgrimages (Hajj and Umrah) [45]. The Madinah region (with a population of 2.2 million) is located in the western part of Saudi Arabia (25°00’N 39°30’E). The capital city, Medina, considered the second-holiest city in Islam, attracts more than seven million annual visitors from all over the world [46]. In addition, samples were also collected from five areas (Al-Shuqaiq, Al-Qahma, Al-Quoz, Al-Qunfudhah and Al-Lith) located along the coastal inhabited area. This area (referred to as the Sahil region in this study) stretches ~ 400 km along the Red Sea coast, linking Jazan with Makkah and Jeddah.

### Mosquito sampling and taxonomic identification

*Aedes aegypti* larvae and eggs were collected between 2019 and 2021 from the five selected regions of Saudi Arabia. All larvae were maintained at 28 ± 2 °C with relative humidity of 75 ± 10%, as previously described for adult emergence [47]. The emerged adults were identified morphologically to species level using a mosquito taxonomic key [48]. For comparison, samples were also included from Southeast Asia (Thailand and Myanmar) and Africa (Uganda). The Chiang Mai, Thailand samples were collected in 2017–2018, and the samples from the three regions in Myanmar were collected in 2004–2005 (Additional file 1: Table S2) [49]. Ugandan *Ae. aegypti* DNA from 2012 and samples from 2020 were used to enable comparison with the ancestral African variation [50].

### DNA extraction and polymerase chain reaction (PCR) amplification

Specimens of *Ae. aegypti* from Saudi Arabia were identified and preserved in tubes with silica gel. Genomic DNA was extracted using the DNeasy Blood and Tissue Kit (QIAGEN Sciences, Germantown, MD, USA). Overall, genomic DNA from a total of 267 adult mosquitoes from Saudi Arabia, Thailand, Myanmar and Uganda was used in this study.

The protocol for the PCR amplification was adapted from Yanola et al. [29]. Two regions in the *vgsc* of the *Ae. aegypti* Saudi strain were amplified using PCR primers: domain IIS6 in VGSC IIP–IIS6 F: GGTGGAACTTCACCGACTTC and R: GGACGCAATCTGGCTTGTTA; and domain IIIS6 in VGSC IIIS4–IIIS6 F: GCTGTCGCACGAGATCATT and R: GTTGAACCCGATGAACAACA. The first primer pair targeted a region containing amino acid positions 989 and 1016, and the second primer pair targeted the region containing position 1534. The PCR reactions were carried out using 5 μl of 5× Q5 reaction buffer, 0.5 μl of 10 mM dNTPs, 1.25 μl (10 μM) of forward and reverse primers, 0.25 μl of 5Q high-fidelity DNA polymerase (New England Laboratory, UK), 2 μl of the DNA template and molecular-grade water to a final reaction volume of 25 μl. Thermal cycling conditions were an initial denaturation at 98 °C for 30 s and 35 cycles as follows: 98 °C for 10 s, 71 °C for 25 s, 72 °C for 25 s) and a 2-min final extension step at 72 °C. The second region was amplified under the same cycling conditions, except that the annealing temperature was 68 °C for 25 s. For the African samples, it was necessary to lower the annealing temperature to 68 °C for successful amplification of domain IIS6, which we suspected was due to sequence variation within the primer-binding regions. Agarose gel electrophoresis (1% agarose gel run for 45–50 min at 100 V) was used to check the quantity and size of the DNA fragments produced. The PCR purification kit (New England Biolabs) was used for amplicon purification prior to Sanger sequencing.

### Sequencing

PCR products were sequenced using the amplification primers (Eurofins Genomics, Germany). Sequence data quality was checked and edited using Geneious Prime software (version 2020.0.3, Biomatters Ltd.) [51]. The resulting sequences were aligned and compared with the *Ae. aegypti* reference sequence from VectorBase (AaegL5.0 Transcript: AAEL023266; Liverpool strain). Following convention, *kdr* mutations are numbered with reference to the homologous house fly *Musca domestica vgsc*. Due to the 233-base-pair (bp) intron in domain IIS6, between the S989P and V1016G mutations, some PCR products were cloned when sequences could not be read from direct sequencing of the PCR product (see the method in Additional file 1: Text S1).

### Genetic diversity analysis

Haplotypes were inferred from the sequenced genotypes using DnaSP (v.6.12.03) [52]. The Population Analysis with Reticulate Trees (PopArt) software (v.4.8.5.) was used to construct median-joining (MJ) haplotype networks to display the putative evolutionary relationships amongst the *kdr* sequences [53]. The method of MJ haplotype network construction was developed to combine features of two algorithms: the heuristic algorithm (Farris's maximum-parsimony), which is used to raise new vertices sequentially known as median vectors, and Kruskal's, which favours short connections to find minimum spanning trees [54]. Haplotype diversity was estimated using DnaSP (v.6.12.03) [52].

## Results

### Detection of *kdr* mutations in the *vgsc*

Sequencing of domains IIS6 and IIIS6 of *vgsc* in 226 individual specimens of *Ae. aegypti* in Saudi Arabia revealed the presence of three *kdr* mutations: S989P and V1016G (domain IIS6) and F1534C (domain IIIS6) due to the substitutions of TCC → CCC, GTA → GGA and TTC → TGC, respectively. The S989P and V1016G mutations are close to each other (~ 311 bp) in domain IIS6 on the sequence alignment and are inferred to be in complete linkage disequilibrium (LD). These mutations varied in frequency among regions (Fig. 1 and Table 1). For S989P and V1016G, the frequency was highest (0.64) in Jeddah and lowest in Sahil (0.28) and Jazan (0.29). For S989P and V1016G, the frequency was low in Myanmar (0.23) and completely absent from Uganda. The frequency of F1534C ranged between 0.56 in Madinah and 0.62 in Sahil (Fig. 1 and Table 1). This mutation was also found in Thailand and Myanmar but was absent from Uganda.

**Table 1:** The frequency of *kdr* mutations (S989P + V1016G and F1534C) in *Aedes aegypti* from Saudi Arabia compared with Southeast Asia

Domain	Codon	Nucleotide	AA	Designation	Saudi Arabia					Southeast Asia	
					Jazan (*n* = 71)	Sahil (*n* = 26)	Jeddah (*n* = 36)	Makkah (*n* = 28)	Madinah (*n* = 32)	Thailand (*n* = 40)	Myanmar (*n* = 24)
IIS6	989–1016	TCC/TCC-GTA/GTA	S/S-V/V	Homozygous wild type	39	13	9	8	12	17	20
	989–1016	TCC/CCC-GTA/GGA	S/P–V/G	Heterozygous	23	12	8	12	12	16	2
	989–1016	CCC/CCC-GGA/GGA	P/P-G/G	Homozygous mutant	9	1	19	8	8	7	2
				Frequency of *kdr* allele (95% CI)	0.29 (0.139—0.343)	0.28 (0.124—0.480)	0.49 (0.462—0.787)	0.50 (0.311—0.689)	0.44 (0.268—0.621)	0.38 (0.232—0.542)	0.23 (0.033—0.335)
IIIS6	1534	TTC/TTC	F/F	Homozygous wild type	9	6	6	6	8	9	16
	1534	TTC/TGC	F/C	Heterozygous	35	8	21	14	12	17	7
	1534	TGC/TGC	C/C	Homozygous mutant	27	12	9	8	12	14	1
				Frequency of *kdr* allele (95% CI)	0.63 (0.441—0.679)	0.63 (0.407—0.790)	0.54 (0.370—0.705)	0.54 (0.342—0.711)	0.56 (0.379—0.731)	0.56 (0.398—0.715)	0.19 (0.066—0.404)

*AA* Amino acid, *S* Serine, *V* Valine, *P* Proline, *G* Glycine, *F* Phenylalanine, *C* Cysteine, *CI* Confidence intervals

Overall, the exon regions of domains IIS6 and IIIS6 were conserved (consistent with worldwide populations of *Ae. aegypti* [36]). No other known *kdr* mutations (i.e. G923V, L982W, I1011M/V, L1014F/S and V1016I) were observed. Mutation F1534S was observed in one individual of the Thailand collection. Four synonymous mutations were found with a relatively high frequency in Saudi Arabia. The first mutation was L971L (TTG → TTA) in domain IIS6 in Saudi Arabia (*n* = 75) and Southeast Asia (*n* = 29) (Additional file 1: Table S3). L971L was only found on haplotypes carrying *kdr* mutations S989P and V1016G (see haplotype network and genetic diversity section for details). Furthermore, the samples that were heterozygous for positions 989 and 1016 (domain IIS6) indicate that mutations S989P and V1016G are associated with a different intron length from the wild type. The other three synonymous mutations were in domain IIIS6 (F1518F, E1591E and G1567G), but were only observed in Jazan and Sahil. Numerous low-frequency non-synonymous substitutions are present in domains IIS6 and IIIS6 in Jazan, Sahil and Uganda, the details of which are shown in Additional file 1: Table S3.

### Genotypes and haplotype frequencies

**Fig. 2:**
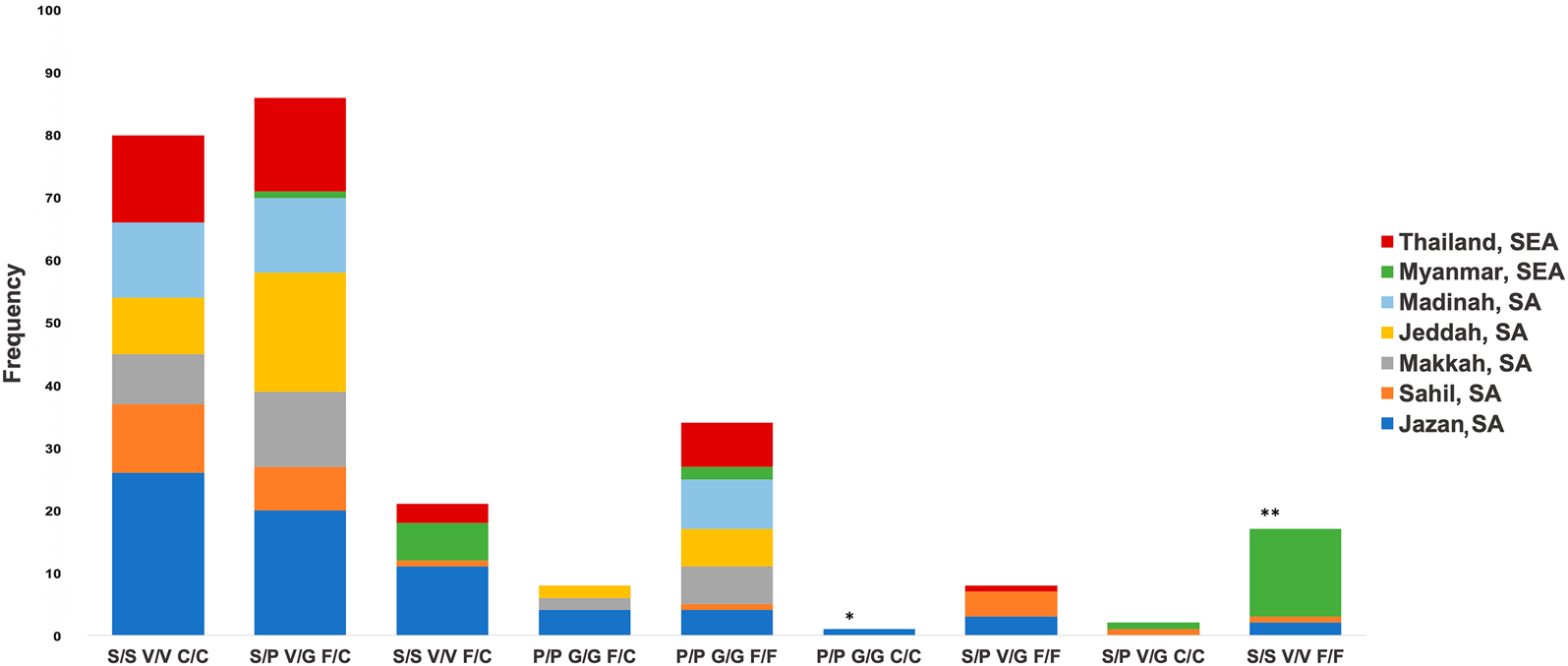
Genotype frequencies of *kdr* mutations S989P + V1016G and F1534C in *Aedes aegypti* from five regions in Saudi Arabia compared with samples from Southeast Asia (Thailand and Myanmar). The bar with one asterisk (*) is the triple homozygous mutant (PP, GG and CC), and the bar with two asterisks (**) is the triple homozygous wild type (SS, VV and FF)

**Fig. 3:**
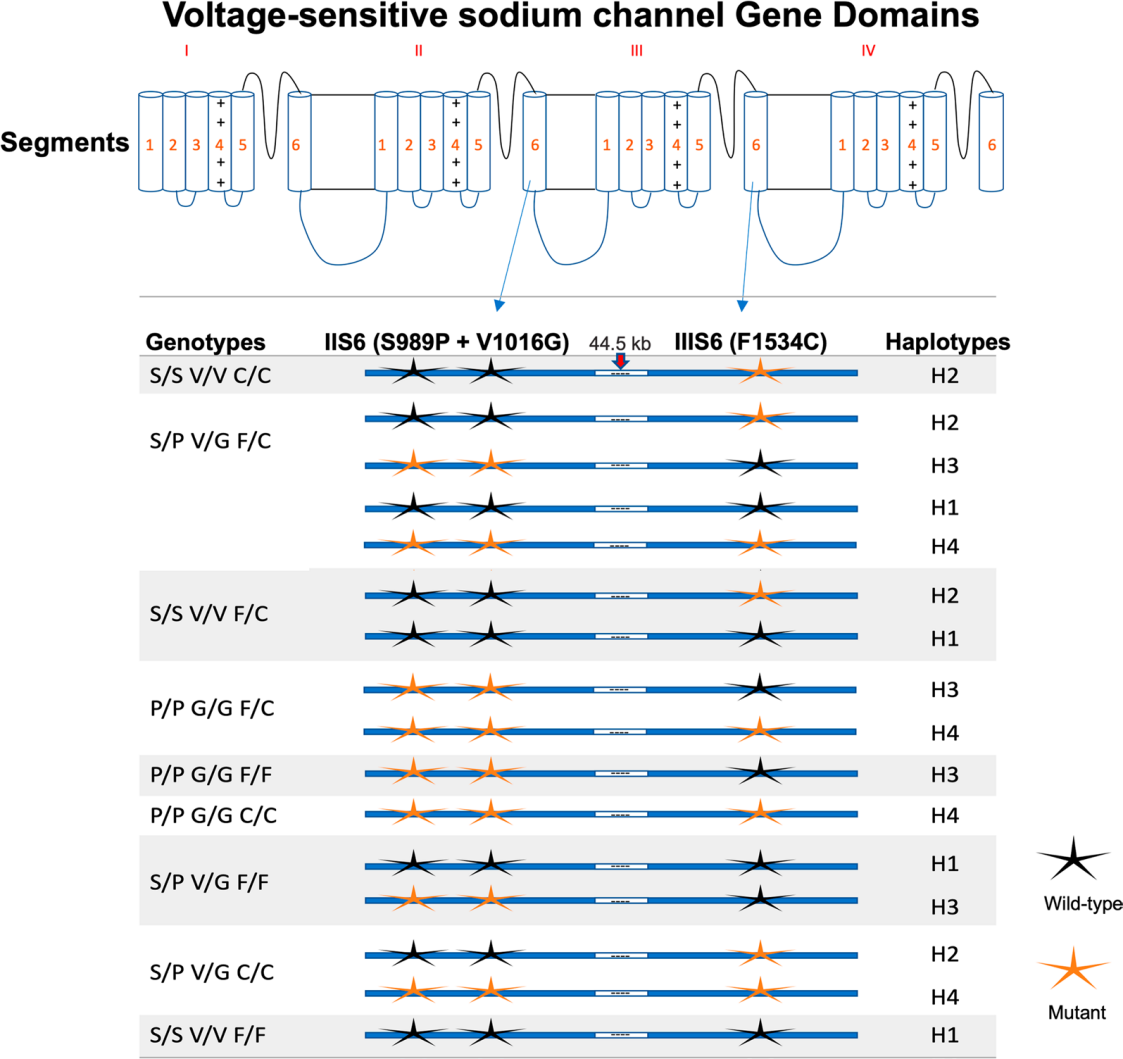
*Kdr* genotypes in *Aedes aegypti* observed from five regions in Saudi Arabia and their possible constituent haplotypes. SS, VV and FF are homozygous wild types for S989P, V1016G and F1534C. PP, GG and GG are homozygous mutants for S989P, V1016G and F1534C. SP, VG and FC are heterozygotes for S989P, V1016G and F1534C. *H1* triple wild type (SVF), *H2* wild types in 989 + 1016 and mutant at 1534 (SVC), *H3* mutant at 989 + 1016 and wild type at 1534 (PGF), *H4* triple mutants (PGC)

A total of nine genotypes involving the three *kdr* mutations S989P, V1016G and F1534C were detected in Saudi Arabia (Fig. 2). These nine genotypes were inferred to comprise four haplotypes with respect to these *kdr* mutations (Fig. 3). Haplotype 1 (H1) is the wild type carrying no known *kdr* mutations; haplotype 2 (H2) carries the F1534C mutation in domain IIIS6; haplotype 3 (H3) is the opposite of H2 and carries S989P and V1016G mutations in domain IIS6; finally, haplotype 4 (H4) is a triple mutant with these *kdr* mutations in both domains IIS6 and IIIS6 (Fig. 3). The triple heterozygote genotype (SP + VG + FC) could theoretically be composed of H1 with H4 or H2 with H3 (Fig. 3). Based on negative linkage disequilibrium observed previously in Saudi Arabia, where mutations S989P and V1016G occur on an alternative haplotypic background to F1534C [17], the triple heterozygote genotypes (SP + VG + FC) observed here are most likely composed exclusively, or almost exclusively, of H2 with H3. On this basis, we estimate haplotype totals of H1 (25), H2 (215), H3 (135), and H4 (11) in our Saudi Arabian samples.

The triple heterozygote genotype (SP + VG + FC) and the genotype containing the homozygous F1534C mutation (SS + VV + CC) were the most frequent genotypes overall in the Saudi Arabia regions sampled (Fig. 2). These genotypes were also commonly observed in Thailand but not in Myanmar, where only one individual had the triple heterozygous genotype (Fig. 2). In this study, a triple homozygous wild genotype (SS + VV + FF) was observed in Jazan (*n* = 2) and Sahil (*n* = 1). This genotype was common in the Myanmar samples (*n* = 14) collected from 2004 to 2005. The genotype that was homozygous for all three *kdr* mutations (PP + GG + CC) was also observed in this study, but only in Jazan (*n* = 1).

### Haplotype network and genetic diversity

**Fig. 4:**
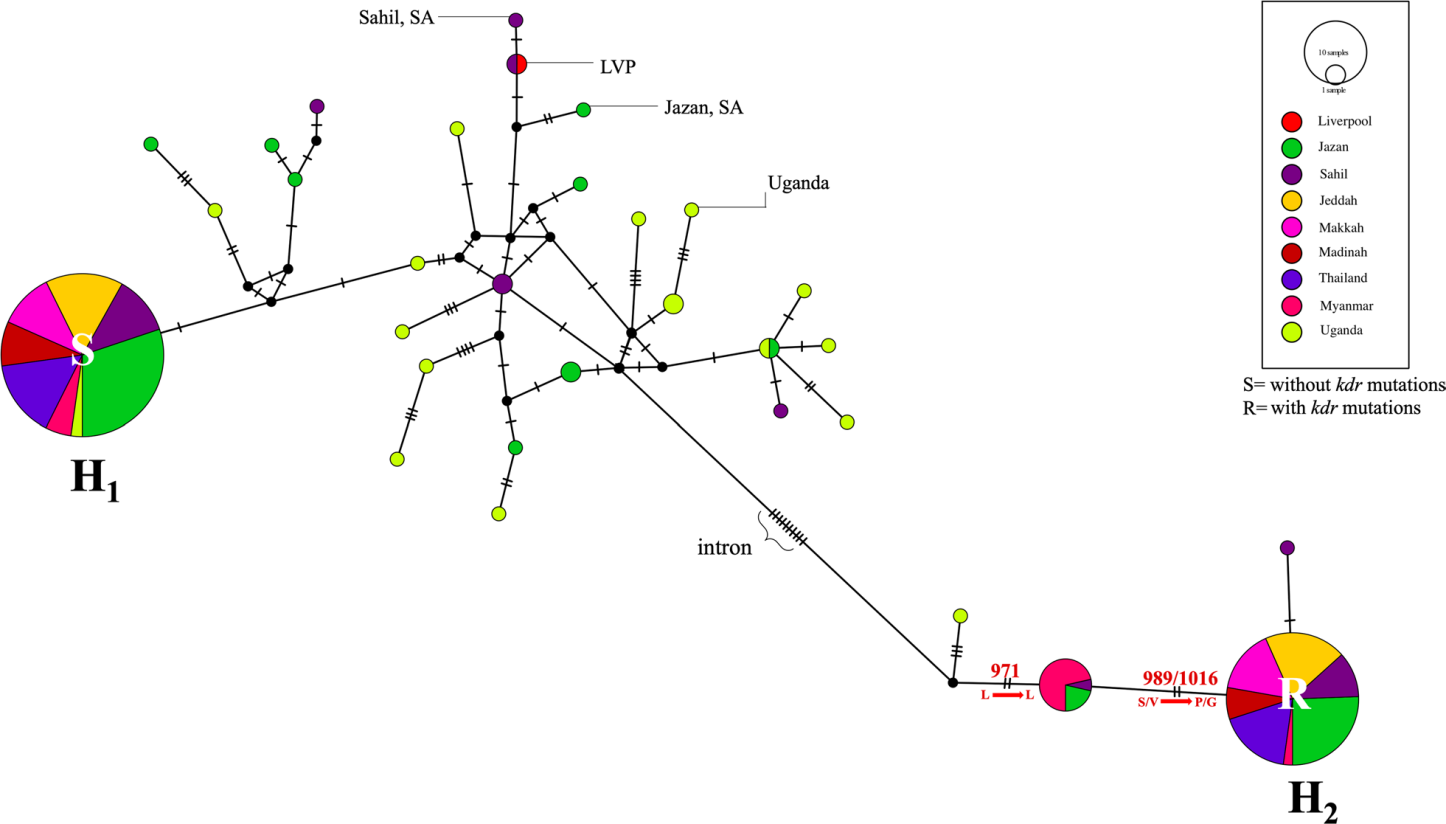
Median-joining haplotype network for domain IIS6 of *vgsc* for Saudi Arabia, Uganda, Thailand and Myanmar populations of *Aedes aegypti*. The coloured circle represents the haplotype and the population. Haplotypes are connected according to their similarity, and hatch marks between haplotypes show the number of mutations. *H* haplotype, *LVP* Liverpool strain

Sequence alignment showed large indels (insertions and deletions) in the intron of domain IIS6 (233 bp) and several in the IIIS6 region (only Jazan and Sahil, along with Uganda). The variation in introns of domain IIS6, particularly between exon 20 (which carries S989P) and exon 21 (carrying V1016G), was particularly large. Indels were frequent in the heterozygotes of S989P + V1016G. In addition, two types of introns in domain IIS6 were observed that differed by ~ 16 bp (corresponding to clade A and B introns), with clade A associated with the mutations in domain IIS6 (S989P + V1016G) (Fig. 4).

**Fig. 5:**
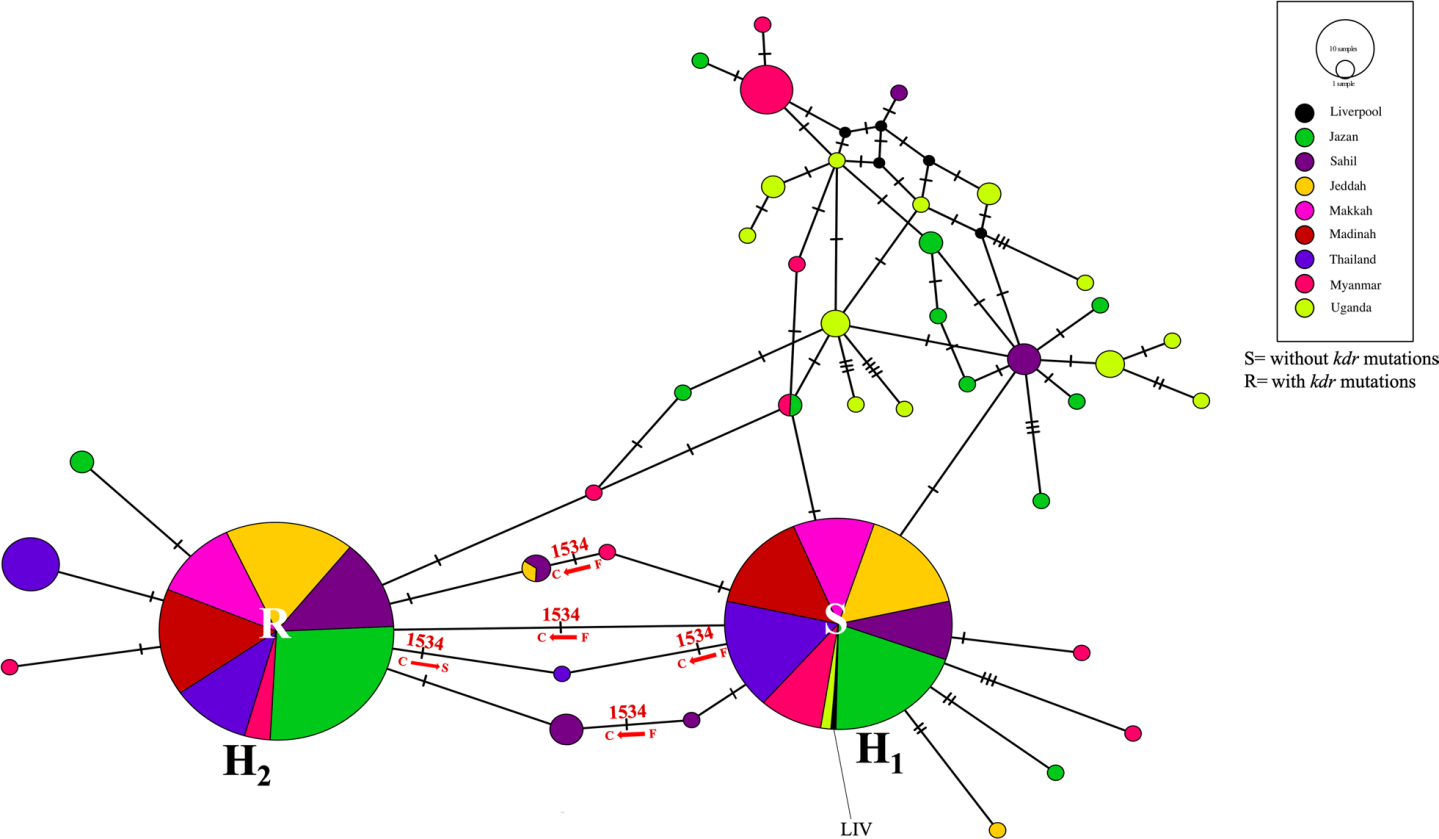
Median-joining haplotype network analysis for domain IIIS6 of *vgsc* for Saudi Arabia, Uganda, Thailand and Myanmar populations of *Aedes aegypti*. The coloured circle represents the haplotype and population. Haplotypes are connected according to their similarity, and hatch marks between haplotypes show the number of mutations. *H* haplotype, *LVP* Liverpool strain

A total of 275 and 468 haplotype sequences of domains IIS6 and IIIS6, respectively, from this study, were aligned and used to construct networks representing the evolutionary relationships amongst the haplotypes (sequence alignments of domain IIS6 and IIIS6 showing polymorphism amongst the haplotypes are shown in Additional file 1: Fig. S3). The networks shown in Figs. 4 and 5 include only the sequences generated in this study using 456 bp and 459 bp from domains IIS6 and IIIS6, respectively. For both domains, haplotypes carrying the resistance mutations occurred at high frequency and were geographically widespread. In each haplotype network, there was also a high-frequency, geographically widespread haplotype that was susceptible (i.e. carrying no *kdr* mutations for that domain). We infer this high frequency due to its carrying *kdr* mutation(s) on the alternative domain to which it is physically linked. In both the IIS6 and IIIS6 networks, these high-frequency haplotypes were the only ones present in the Makkah, Jeddah and Madinah regions of Saudi Arabia, and Thailand, consistent with their high levels of *kdr* mutations. Conversely, there are many low-frequency haplotypes from the Jazan and Sahil regions of Saudi Arabia in both networks, where they intermingle with a large number of diverse haplotypes from Uganda, as well as some haplotypes from Myanmar. This is reflected in the high haplotype diversity for Jazan, Sahil and Uganda for both domains IIS6 and IIIS6 (Table 2).

**Table 2:** Haplotype diversity in domains IIS6 and IIS6 in the eight study populations

Domain/parameter	Saudi Arabia					Southeast Asia		Africa
	Jazan	Sahil	Jeddah	Makkah	Madinah	Thailand	Myanmar	Uganda
Domain II								
Haplotype-diversity (Hd)	0.62	0.70	0.51	0.52	0.49	0.50	0.61	0.95
Number of haplotypes	11	9	2	2	2	2	3	9
Domain III								
Haplotype-diversity (Hd)	0.61	0.69	0.53	0.51	0.51	0.64	0.77	0.95
Number of haplotypes	13	8	4	2	2	4	11	13

Our sequence data were also combined with haplotypes from 40 populations retrieved from GenBank to generate networks [36] (Additional file 1: Figs. S1, S2). This utilised a total of 483 and 590 homologous sequences that were 173 and 314 bp in length for domains IIS6 and IIIS6, respectively. Overall, there were 57 haplotypes (haplotype diversity: 0.758) in domain IIS6 and 45 haplotypes (haplotype diversity: 0.657) in domain IIIS6 (Additional file 1: Figs. S1, S2).

## Discussion

Dengue fever is endemic in the five geographical regions of southwestern Saudi Arabia studied here, namely, Jazan, Sahil, Jeddah, Makkah and Madinah [55]. This study concurrently characterised *kdr* mutations in these dengue-endemic regions of Saudi Arabia by sequencing domains IIS6 and IIIS6 of *vgsc* and comparing them with the variation found in samples from Thailand, Myanmar and Uganda. The *kdr* mutations S989P, V1016G and F1534C have previously been reported from Makkah and Jeddah [18, 35, 56] and from a laboratory strain of *Ae. aegypti* from Jazan [57]. This is the first report of *kdr* mutations S989P, V1016G and F1534C from natural populations in Sahil, Madinah and Jazan.

The higher frequency of the S989P and V1016G *kdr* mutations in the cities of Jeddah, Makkah and Madinah compared with the more rural areas of Sahil and Jazan indicates differences in insecticide application and selection pressures between urban and rural areas. Given the high levels of resistance to both type I and type II pyrethroids due to the genotype S989P + V1016G [25], we suggest that this has resulted from higher levels of insecticide usage, of both thermal and ultra-low-volume fogging, and household sprays (Additional file 1: Table S4), in urban relative to rural areas. Other studies have similarly found higher levels of resistance in urban compared with rural areas, i.e. in Indonesia [58], Malaysia [59] and Tunisia [60].

In all studied areas of Saudi Arabia, the F1534C mutation was also present at high frequency. As F1534C is almost always located on a different haplotypic background from S989P + V1016G, the highest frequency possible of the triple heterozygous genotype is ~ 0.5, resulting from underlying allele frequencies of ~ 0.5 for both S989P + V1016G and F1534C. The triple heterozygous *kdr* genotype (SP + VG + FC) is at high frequency throughout Saudi Arabia, reaching the highest possible frequency in Jeddah and Makkah. This concurs with a recent report on the high frequency of the triple heterozygote genotype in Jeddah [35]. Notably, very similar allele frequencies for these *kdr* mutations and the triple heterozygote genotype have also been observed in Chiang Mai, Thailand [38], with their temporal stability suggested as being maintained by balancing selection [61]. Given that the double homozygous S989P + V1016G genotype, i.e. PP + GG + FF, has high levels of resistance to both type I and type II pyrethroids [25], balancing selection is most likely due to increased fitness of the triple heterozygous genotype (SP + VG + FC) in the absence of insecticidal selection pressure, as was demonstrated by Saingamsook et al. [62].

As previously seen in Saudi Arabia and elsewhere [33, 36, 56, 63], we observed that *kdr* mutations S989P and V1016G in our study were linked to the clade A intron type. The haplotype network for our study populations, as well as other worldwide populations (Fig. 4, Additional file 1: Table S3; Fig. S1), indicates that these *kdr* mutations occurred on the haplotypic background carrying not only the clade A intron type but also the synonymous mutation L971L, indicating a single evolutionary origin of these *kdr* mutations, as previously inferred [36].

The F1534C *kdr* mutation has previously been inferred to have at least two independent evolutionary origins [36]. Our haplotype network for domain IIIS6, which contains this mutation, shows extensive reticulation in the network involving primarily synonymous mutations from Uganda and Saudi Arabia but also F1534C (Fig. 5 and Additional file 1: Fig. S2). The reticulation indicates either recombination or homoplasy, i.e. repeated mutations at the same site, but the latter seems more likely given the limited length of this region. We cannot exclude the possibility of F1534C arising more than once, but it is possible to explain all the data with a single evolutionary origin of the widespread haplotype containing F1534C coupled with repeated mutations at synonymous sites.

We found in this study one individual from Thailand with a non-synonymous substitution to 1534S. This could have arisen from the susceptible background, i.e., by mutation F1534S or from the background carrying the *kdr* mutation, i.e., by mutation C1534S. F1534S has been reported as a *kdr* mutation in *Ae. albopictus* collected from the United States and China [64, 65] and its resistance status in *Ae. aegypti* should be tested.

Interestingly, this study reported one individual with a triple homozygous mutant genotype (PP + GG + CC) from the Jazan region, and the triply mutant haplotypes can also be inferred to be present in Jazan, Jeddah, Makkah and Sahil (from Figs. 2 and 3). This genotype has previously been reported at low frequency in Jeddah [35, 56], Myanmar [30] and Laos [66]. Given the large intron (> 44.5 kb) between domains IIS6 and domain IIIS6, the triple mutant haplotype carrying all *kdr* mutations could likely have arisen due to recombination. This genotype provided an extremely high resistance level to pyrethroid insecticides when artificially introduced into *Xenopus* oocytes [40]. It may therefore be favoured by insecticide pressure, but its low frequency in natural populations suggests a high fitness cost in the absence of insecticide application. A major concern is that secondary mutations may arise that mitigate this fitness cost, which could then result in extremely high levels of pyrethroid resistance in Saudi Arabia. Continuous surveillance for this triply mutated haplotype, the triple homozygous mutant genotype and potential fitness-restoring mutations is a high priority.

A high frequency (~ 58%) of fully susceptible wild type genotypes (SS + VV + FF) was detected in this study in samples from Myanmar. They were collected in 2004–2005 when insecticide usage was likely much lower. This fully susceptible genotype was not reported in a more recent study in Myanmar; however, the susceptible haplotype is still present [27, 30]. Previous studies have reported the completely susceptible wild type genotype in the Indo-Pacific, including Taiwan [33], Queensland, Australia [67] and Malaysia [68]. For the first time, this study reported the presence of the triple homozygote wild type genotype in two individuals from the highlands of Jazan and the Al-Quoz governorate of Sahil, where it is most likely that insecticides have been less intensively applied than in the large cities. However, we can also infer the presence of the wild type haplotypes (S + V + F) from Jeddah and Makkah (from Figs. 2 and 3). In Saudi Arabia, the susceptible haplotype was observed only in Jeddah previously [18]. This indicates the possibility of restoring susceptibility to pyrethroids through a well-designed public health pest management strategy in Saudi Arabia.

Finally, for the first time, this study reported exceptionally high levels of genetic diversity in the Sahil and Jazan populations at wild type alleles that are comparable to African levels of genetic diversity. This implies a direct contribution of African ancestry to Saudi Arabian populations, which we will explore in detail elsewhere (Mashlawi et al. in preparation).

## Conclusion

This study has brought new insight into the population genetics of *kdr* in *Ae. aegypti* populations from Saudi Arabia. The presence of the triple homozygous wild type haplotypes in the studied populations calls for further consideration of insecticide resistance management strategies that could restore sensitivity to pyrethroids in *Ae. aegypti* in Saudi Arabia. Two predominant genotypes were observed (SP + VG + FC and SS + VV + CC) in *Ae. aegypti* across five regions of Saudi Arabia indicating ongoing selection for resistance to pyrethroids. The genotyping results of specimens from Jazan, Sahil, Jeddah, Makkah and Madinah can be used as baseline information for a potential future trial of the *Wolbachia*-infected *Ae. aegypti* method for dengue control due to the requirement of homogeneity of *kdr* genotypes between field and released strains [35]. Long-term high levels of *Ae. aegypti* resistance to pyrethroids should not be considered inevitable. For example, although pyrethroid insecticides have been applied in Queensland, Australia since the 1990s in response to dengue fever outbreaks, a strategy of integrated vector control methods has delayed the emergence of resistance and as of 2017, there has been no evidence of *kdr* mutations [67]. An additional success story of integrated vector management is the case of Singapore, in which the integration of effective tools and approaches, improved surveillance, and community engagement were keys to success [69]. Integrated vector control using multiple approaches and alternatives to insecticides should be considered in Saudi Arabia. The presence of wild type haplotypes in Saudi Arabia carrying no *kdr* mutations offers the possibility to restore susceptibility that could allow improved efficacy of insecticides as part of an integrated vector control strategy.

## Supplementary Information

**Additional file 1: Table S1.** Localities/Districts, region, number of sites, coordinates and elevation (m = meter above the sea level) of collections of *Aedes aegypti* in Saudi Arabia. **Table S2** Countries, regions, coordinates and collection year of *Aedes aegypti* in Southeast Asia. **Table S3.** Four synonymous mutations observed in domains IIS6 and IIIS6 of *Aedes aegypti* in Saudi Arabia and Southeast Asia. **Table S4.** A summary of vector control strategies and insecticide spraying regimes in major cities of Saudi Arabia, Thailand, Myanmar and Uganda. **Text S1.** DNA cloning protocol. **Figure S1.** Median-joining haplotypes network analysis for domain IIS6 of *vgsc* in 40 populations of *Aedes aegypti* worldwide. The original haplotype networks are with notes. Circles with colour represent the haplotype and population. Haplotypes are connected according to their similarity, and hatch marks between haplotypes show the base-pair mutations. S = susceptible; R = resistant. **Figure S2.** Median-joining haplotypes network analysis for domain IIIS6 of *vgsc* in 27 populations of *Aedes aegypti* worldwide. The original haplotype networks are with notes. Circles with colour represent the haplotype and population. Haplotypes are connected according to their similarity, and hatch marks between haplotypes show the base-pair mutations. **Figure S3.** The SNP sites identified in each domain to infer the haplotype network. **A** is for domain IIS6 and **B** is for domain IIIS6. Red squares indicate the non-synonymous mutations (*kdr*) in each domain. These are haplotype networks amongst our sequences.

## Data Availability

The sequences haplotype result for IIS6 and IIIS6 in *vgsc*, 498 bp and 567 bp, respectively, are available from NCBI GenBank (Accession for domain IIS6: ON651284-ON651417 and ON651284-ON651364 for domain IIIS6) and Dryad Dataset at https://datadryad.org/stash/share/wDIWDnXcz-KFbGRmvrMhcsqBZiB37u2YF_Qa3lToFx8

## Abbreviations

*kdr*: Knockdown resistance
VGSC: Voltage-gated sodium channel
MJ: Median-joining
