# Supplementary material for: Molecular analysis of knockdown resistance (kdr) mutations in the voltage-gated sodium channel gene of Aedes aegypti populations from Saudi Arabia
Source: Parasit Vectors. 2022 Oct 19;15:375. doi: 10.1186/s13071-022-05525-y (PMC9583590; doi:10.1186/s13071-022-05525-y)
Supplement: Supplementary file 1 — Additional file 1: Table S1. Localities/Districts, region, number of sites, coordinates and elevation (m = meter above the sea level) of collections of Aedes aegypti in Saudi Arabia. Table S2 Countries, regions, coordinates and collection year of Aedes aegypti in Southeast Asia. Table S3. Four synonymous mutations observed in domains IIS6 and IIIS6 of Aedes aegypti in Saudi Arabia and Southeast Asia. Table S4. A summary of vector control strategies and insecticide spraying regimes in major cities of Saudi Arabia, Thailand, Myanmar and Uganda. Text S1. DNA cloning protocol. Figure S1. Median-joining haplotypes network analysis for domain IIS6 of vgsc in 40 populations of Aedes aegypti worldwide. The original haplotype networks are with notes. Circles with colour represent the haplotype and population. Haplotypes are connected according to their similarity, and hatch marks between haplotypes show the base-pair mutations. S = susceptible; R = resistant. Figure S2. Median-joining haplotypes network analysis for domain IIIS6 of vgsc in 27 populations of Aedes aegypti worldwide. The original haplotype networks are with notes. Circles with colour represent the haplotype and population. Haplotypes are connected according to their similarity, and hatch marks between haplotypes show the base-pair mutations. Figure S3. The SNP sites identified in each domain to infer the haplotype network. A is for domain IIS6 and B is for domain IIIS6. Red squares indicate the non-synonymous mutations (kdr) in each domain. These are haplotype networks amongst our sequences. [file 13071_2022_5525_MOESM1_ESM.pdf]

Supplementary file

Molecular analysis of *knockdown resistance* (*kdr*) mutations in *Aedes aegypti* populations from Saudi Arabia

Abadi M. Mashlawi[1,2,*], Ashwaq M. Al-Nazawi[3], Elsiddig M. Noureldin[4], Hussain Alqahtani[1,5], Jazem A. Mahyoub[6], Jassada Saingamsook[7], Mustapha Debboun[8], Martha Kaddumukasa[9], Hesham M. Al-Mekhlafi[10,11] and Catherine Walton[1,*]

Additional file 1.

Table S1 Localities/ Districts, region, number of collection sites, coordinates, and elevation (m=meter above the sea level) of collections of *Aedes aegypti* in Saudi Arabia

Region	Localities/ District	Sites (No.)	Coordinates		Elevation (m)
			N	E	
Jazan	Sabya	12	17°09'04.32"	42°40'59.97"	59
Jazan	Ad Darb	5	17°43'46.47"	42°15'06.17"	69
Jazan	Jazan City	5	16°55'56.70"	42°33'47.74"	19
Jazan	Gizan Highland	8	17°14'00.89"	42°56'28.72"	1327
Jazan	Abu Arish	2	16°58'10.55"	42°50'02.37"	75
Sahil	Al Shuqaiq	4	17°43'35.86"	42°01'45.67"	40
Sahil	Al Qahma	2	18°00'12.30"	41°40'33.46"	2
Sahil	Al Quoz	5	18°58'16.99"	41°19'21.25"	62
Sahil	Al Qunfudhah	3	19°07'26.71"	41°05'12.32"	9
Sahil	Al Lith	2	20°09'07.11"	40°16'44.11"	5
Makkah	Al Kakiyyah	2	21°23'01.7"	39°49'01.2"	281
Makkah	Alawali	17	21°21'10.8"	39°53'16.4"	259
Makkah	Al Shuhada	2	21°38'24.4"	39°42'53.4"	214
Makkah	At Taniem	2	21°28'48.7"	39°47'45.6"	280
Makkah	Batha Quraish,	1	21°22'35.8"	39°49'48.7"	253
Jeddah	Al Muntazahat	1	21°27'40.38"	39°17'14.94"	61
Jeddah	Ath Thaalibah	1	21°27'44.62"	39°11'30.59"	2
Jeddah	Ghulail	7	21°27'04.82"	39°11'51.97"	4
Jeddah	Mada'en Al-Fahd	2	21°27'33.12"	39°15'27"	33
Jeddah	Al Mohammadiyyah	2	21°38'44.00"	39°07'45.12"	6
Jeddah	Abruq Ar Rughamah	1	21°29'19.92"	39°18'04.63"	90
Madinah	Al Nakeel	1	24°28'12.4"	39°37'45.1"	616
Madinah	Al Awali	2	24°30'43.0"	39°33'16.2"	629
Madinah	Quba	1	24°24'52.9"	39°34'00.1"	645
Madinah	Al Aqiq	2	24°27'23.4"	39°35'34.4"	622
Madinah	Uhud	1	24°33'06.5"	39°36'35.6"	592

Table S2 Countries, regions, location coordinates, and collection year of *Aedes aegypti* in Southeast Asia and Uganda.

Country	Region	Longitude	Latitude	Collection Year
Thailand	Chiang Mai	18°47'15.8"	98°59'03.3"	2017-2018
Myanmar	Yangon	16°27'00.0"	96°10'12.0"	2004
Myanmar	Meiktila	20°51'00.0"	95°49'48.0"	2005
Myanmar	Myitkyina	25°21'36.0"	97°23'24.0"	2005
Uganda	Entebbe - Zika forest	0°07'17.1"	32°31'34.6"	2020
Uganda	Fort Portal	0°39'00.0"	30°16'12.0"	2012
Uganda	Semuliki forest	0°42'57.6"	30°03'36.0"	2012

Table S3 Four frequent synonymous mutations observed in domain IIS6 and IIIS6 of *Aedes aegypti* in Saudi Arabia and Southeast Asia.

Codon	SNPs	Amino acid	Domain	Populations	Total
**971**	TTG – TTA	L/L	IIS6	Jazan (*n*=26), Sahil (*n*=10), Jeddah (*n*=18), Makkah (*n*=14), Madinah (*n*=7), Thailand (*n*= 16), and Myanmar (*n*=13)	104
**1518**	TTT – TTC	F/F	IIIS6	Jazan (*n*=8) and Sahil (*n*=6)	14
**1591**	GAG – GAA	E/E	IIIS6	Jazan (*n*=6) and Sahil (*n*=1)	7
**1567**	GGT – GGA	G/G	IIIS6	Sahil (*n*=6)	6

Table S4 Summary of vector control strategies and insecticide spraying regimes in major cities of Saudi Arabia, Thailand, Myanmar and Uganda.

Country	Control strategies	Insecticides used	Spraying regimes	References
Saudi Arabia	- IRS<br>- Space spaying: Ultra-low volume (ULV) (also known as cold fogs) and thermal fogging<br>- ITNs or LLINs (practiced since 1999 – 2011)<br>- Outdoor control measures (Source reduction of mosquito breeding sites)	- Pyrethroids<br>- Organophosphate	- Twice daily (morning and evening) in most cities	[1–3]
Thailand	- IRS<br>- ITNs and LLINs<br>- Space spaying: Ultra-low volume (ULV) and thermal fogging<br>- Commercial household aerosol spray containing pyrethroid-based formulations (popular strategy)	- Pyrethroids<br>- Organophosphate	- Spraying within a 100 m^2 radius where the dengue case occurred<br>- Spraying every week until there are no dengue cases in the area	[4–6]
Myanmar	- IRS<br>- LLINs<br>- Space spaying: thermal fogging<br>- Outdoor control measures<br>- Health education	- Pyrethroids (since 1992)<br>- Organochlorine and Organophosphate (1988–2005)	- Spraying within a 100 m^2 radius where the dengue case occurred	[7, 8]
Uganda	- LLINs (universal coverage)<br>- IRS	- Pyrethroids	- LLINs: government distributed free throughout the country; IRS: in selected areas.	[9]

IRS: Indoor residual spraying, ITNs: Insecticide treated bed nets, LLINs: Long-lasting insecticide-treated nets.

References

1. Coleman M, Al-Zahrani MH, Coleman M, Hemingway J, Omar A, Stanton MC, et al. A country on the verge of malaria elimination--the Kingdom of Saudi Arabia. Plos One. 2014;9:e105980.
2. Al-Nazawi AM, Aqili J, Alzahrani M, McCall PJ, Weetman D. Combined target site (*kdr*) mutations play a primary role in highly pyrethroid resistant phenotypes of *Aedes aegypti* from Saudi Arabia. Parasit Vectors. 2017;10:161.
3. Alsheikh A, Mohammed W, Noureldin E, Daffalla O, Shrwani K, Hobani Y, et al. Resistance status of *Aedes aegypti* to insecticides in the Jazan Region of Saudi Arabia. Biosci, Biotech Res Asia. 2016;13:155–62.
4. Nachaiwieng W, Yanola J, Chamnanya S, Lumjuan N, Somboon P. Efficacy of five commercial household insecticide aerosol sprays against pyrethroid resistant *Aedes aegypti* and Culex quinquefasciatus mosquitoes in Thailand. Pestic Biochem Physiol. 2021;178:104911.
5. Srichan P, Niyom SL, Pacheun O, Iamsirithawon S, Chatchen S, Jones C, et al. Addressing challenges faced by insecticide spraying for the control of dengue fever in Bangkok, Thailand: a qualitative approach. Int Health. 2018;10:349-355.
6. Chareonviriyaphap T, Bangs MJ, Suwonkerd W, Kongmee M, Corbel V, Ngoen-Klan R. Review of insecticide resistance and behavioral avoidance of vectors of human diseases in Thailand. Parasit Vectors. 2013;6:280.
7. Zhong D, Aung PL, Mya MM, Wang X, Qin Q, Soe MT, Zhou G, Kyaw MP, Sattabongkot J, Cui L, Yan G. Community structure and insecticide resistance of malaria vectors in northern-central Myanmar. Parasit Vectors. 2022;15:155.
8. Kawada H, Oo SZM, Thaung S, Kawashima E, Maung YNM, Thu HM, et al. Co-occurrence of point mutations in the voltage-gated sodium channel of pyrethroid-resistant *Aedes aegypti* populations in Myanmar. PLoS Negl Trop Dis. 2014;8:e3032.
9. Katureebe A, Zinszer K, Arinaitwe E, Rek J, Kakande E, Charland K, Kigozi R, Kilama M, Nankabirwa J, Yeka A, Mawejje H. Measures of malaria burden after long-lasting insecticidal net distribution and indoor residual spraying at three sites in Uganda: a prospective observational study. PLoS medicine. 2016;13(11):e1002167.

Text S1 ***DNA Cloning Protocol***

PCR products of 4.4µl in a mix with 2µl of 5X GoTaq reaction buffer, 2µl of 1mM dATP (0.2mM final concentration), 1µl of GoTaq Flexi DNA polymerase (5u/µl), 0.6µl of 25mM MgCl2 (1.5mM final concentration) and nuclease-free water to a final volume of 10µl were incubated at 70°C for 30 min to ensure adding the a-tailing end to use T-vector cloning. After the a-tailing reaction, 2µl of the products were used in the standard reaction ligation following the kit protocol of Promega (Promega, Hampshire UK) carried in a total reaction volume of 10µl with 5µl of 2X Rapid Ligation Buffer, T4 DNA Ligase, 1µl of pGEM®-T or pGEM®-T Easy Vector (50ng), 1µl of T4 DNA Ligase (3 Weiss units/µl) and nuclease-free water to a final volume. Positive control and background control reactions were used to ensure the efficiency of ligation. The protocol recommended one-hour ligation; however, overnight ligation was found to increase the transformation efficiency. After that, for transformation, LB/ampicillin/IPTG/X-Gal plates were prepared prior to the transformation and plates were kept at 4°C. Transformation was carried as following: 2µl of over-night ligation was transferred to 1.5 ml Eppendorf tube and kept on ice, 25µl of JM109 Competent Cells, High Efficiency was added and incubated for 20 min on ice, 42°C heat-shock was done for 45-50 seconds and returned immediately to ice for 2 min. 950µl of SOC medium (room temperature) was added. The transformation was incubated for 90 min at 37°C with shaking (~150rpm). Finally, 100µl of each transformation was spread over to double plates. Plates were incubated over-night (16-24h) at 37°C. Each plate was selected with at least three colonies, amplified, and sequenced.

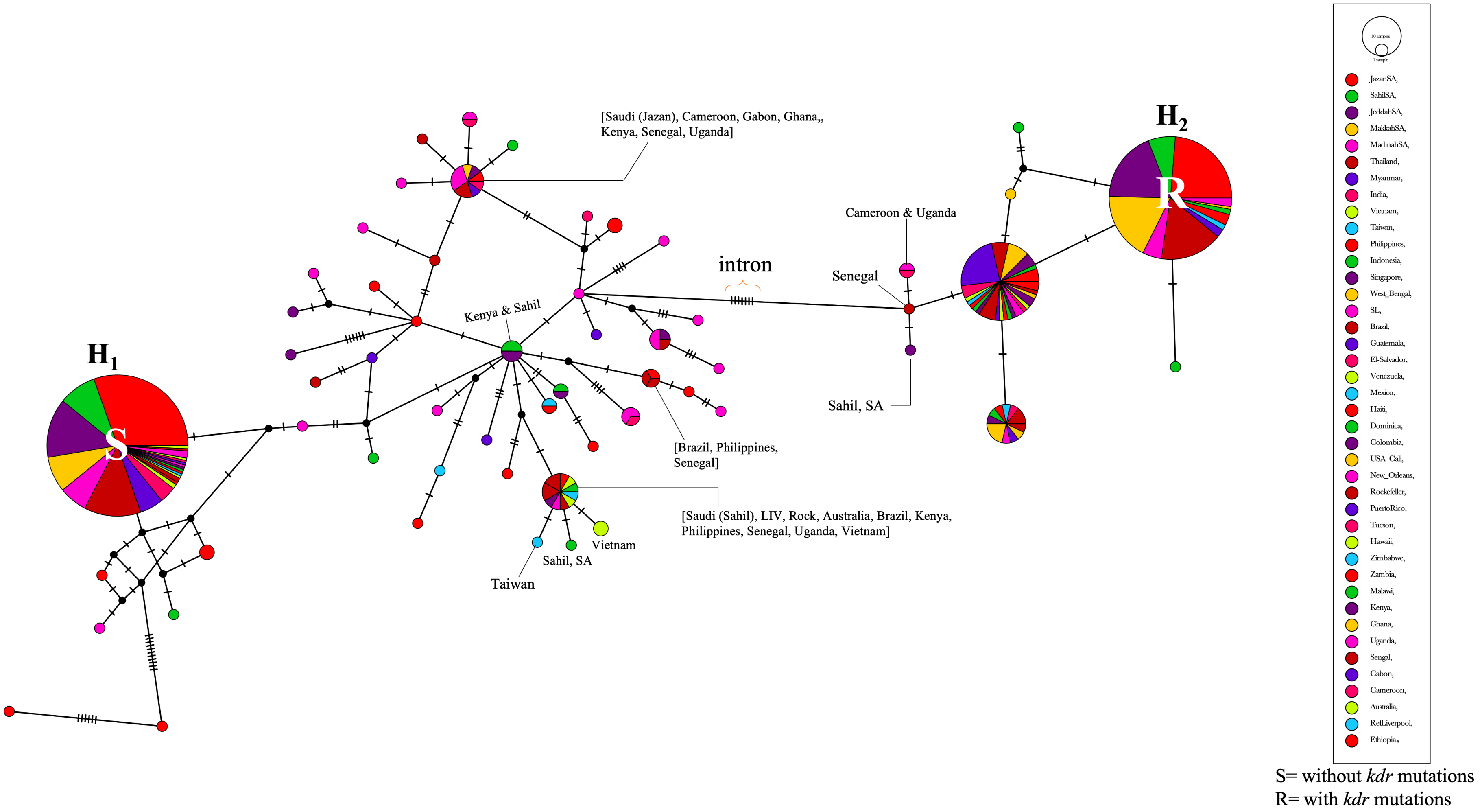

Fig. S1 Median-joining haplotypes network analysis for domain IIS6 of *vgsc* in 40 populations of *Aedes aegypti* worldwide. The original haplotype networks are with notes. Circles with color represents the haplotype and population. Haplotypes are connected according to their similarity, and hatch marks between haplotypes show the base-pair mutations. S= susceptible; R= resistant.

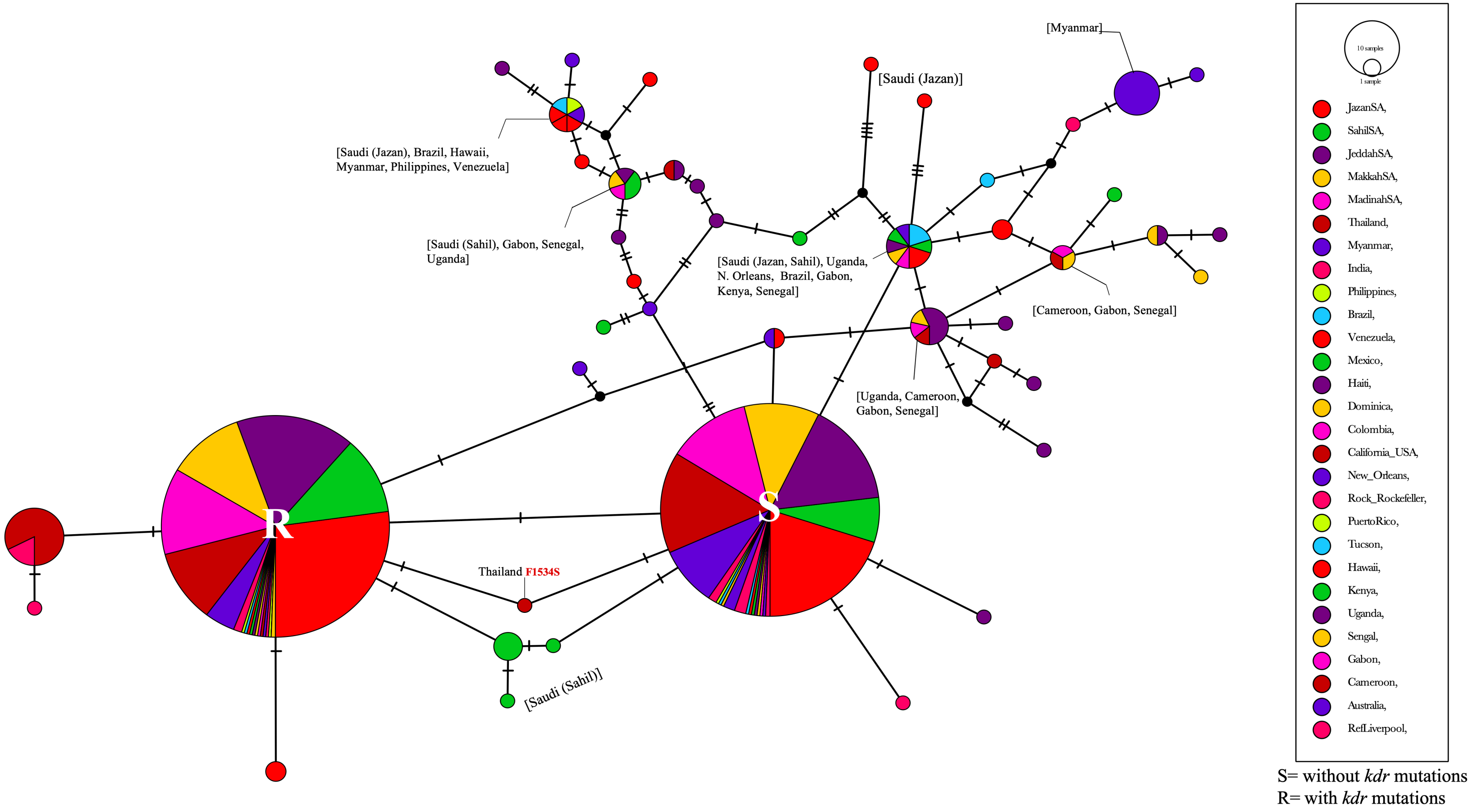

Fig. S2 Median-joining haplotypes network analysis for domain IIIS6 of *vgsc* in 27 populations of *Aedes aegypti* worldwide. The original haplotype networks are with notes. Circles with color represents the haplotype and population. Haplotypes are connected according to their similarity, and hatch marks between haplotypes show the base-pair mutations.

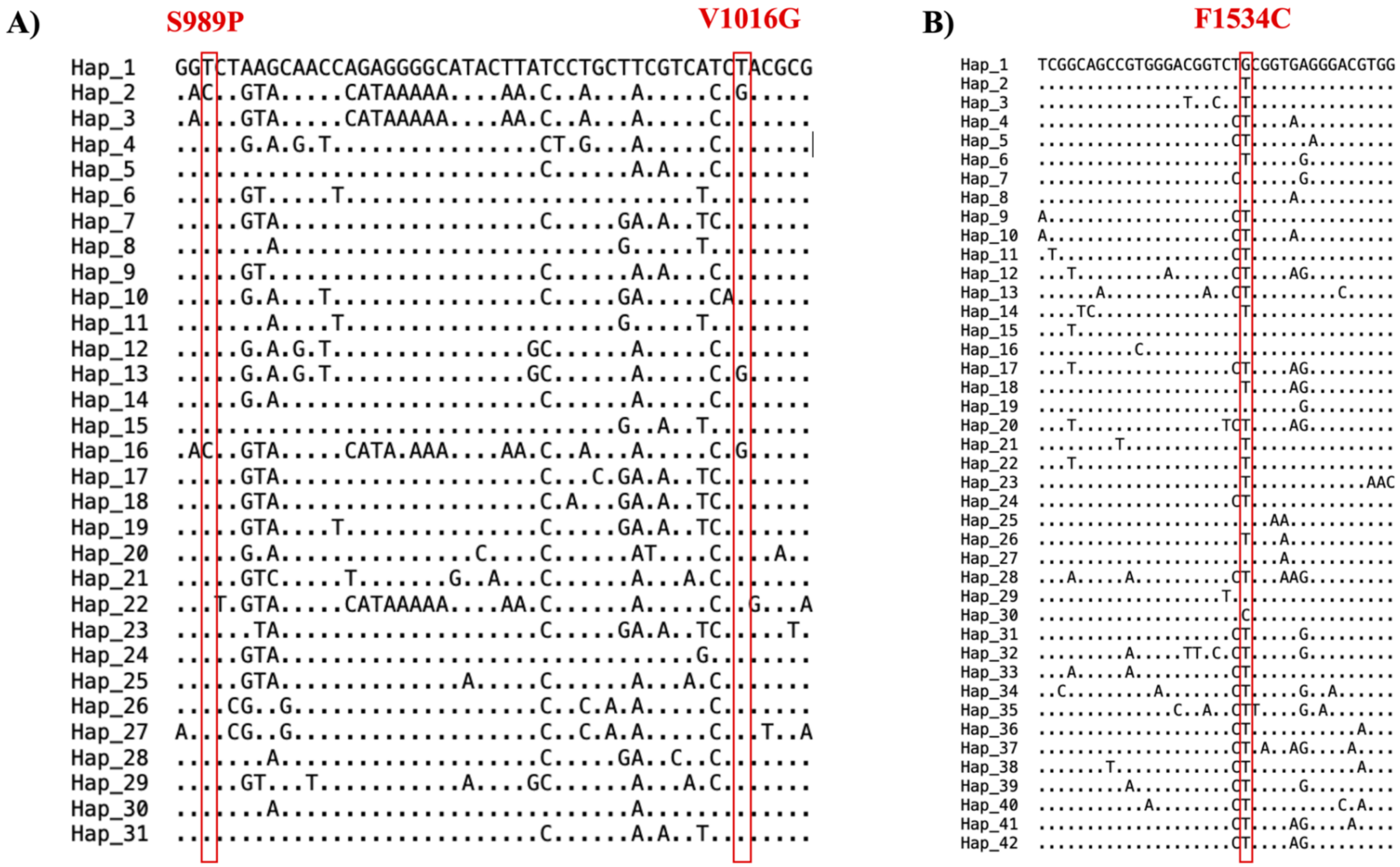

Fig. S3 The SNP sites identified in each domain to infer the haplotype network. **A)** is for domain IIS6 and **B)** is for domain IIIS6. Red squares are the non-synonymous mutations (*kdr*) in each domain. These are haplotype networks amongst our sequences.
